# The Arabidopsis *JAGGED LATERAL ORGANS* (*JLO*) gene sensitizes plants to auxin

**DOI:** 10.1093/jxb/erx131

**Published:** 2017-05-02

**Authors:** Madlen I Rast-Somssich, Petra Žádníková, Stephan Schmid, Martin Kieffer, Stefan Kepinski, Rüdiger Simon

**Affiliations:** 1 Institute for Developmental Genetics, Cluster of Excellence in Plant Sciences (CEPLAS), Heinrich Heine Universität, Universitätstrasse, Düsseldorf, Germany; 2 Centre for Plant Sciences, Faculty of Biological Sciences, University of Leeds, Leeds, UK

**Keywords:** Auxin perception, auxin receptor, *JAGGED LATERAL ORGANS*, *JLO*, *LBD*, root development, *TIR1*/*AFB*

## Abstract

Plant growth and development of new organs depend on the continuous activity of the meristems. In the shoot, patterns of organ initiation are determined by PINFORMED (PIN)-dependent auxin distribution, while the undifferentiated state of meristem cells requires activity of KNOTTED LIKE HOMEOBOX (KNOX) transcription factors. Cell proliferation and differentiation of the root meristem are regulated by the largely antagonistic functions of auxin and cytokinins. It has previously been shown that the transcription factor JAGGED LATERAL ORGANS (JLO), a member of the LATERAL ORGAN BOUNDARY DOMAIN (LBD) family, coordinates *KNOX* and *PIN* expression in the shoot and promotes root meristem growth. Here we show that JLO is required for the establishment of the root stem cell niche, where it interacts with the auxin/PLETHORA pathway. Auxin signaling involves the AUX/IAA co-repressor proteins, ARF transcription factors and F-box receptors of the TIR1/AFB1–5 family. Because *jlo* mutants fail to degrade the AUX/IAA protein BODENLOS, root meristem development is inhibited. We also demonstrate that the expression levels of two auxin receptors, TIR1 and AFB1, are controlled by JLO dosage, and that the shoot and root defects of *jlo* mutants are alleviated in *jlo* plants expressing *TIR1* and *AFB1* from a transgene. The finding that the auxin sensitivity of a plant can be differentially regulated through control of auxin receptor expression can explain how different developmental processes can be integrated by the activity of a key transcription factor.

## Teaser

The transcription factor JLO regulates stem cell niches and plant development by controlling expression of the auxin receptors TIR1 and AFB1.

## Introduction

Unlike animals, whose basic body structure is defined during embryogenesis, plants have the ability to constantly produce new organs from pools of stem cells that are located primarily at the shoot apical meristem (SAM) and root apical meristem (RAM). The RAM consists of a quiescent center (QC) in the center of the root meristem that has low mitotic activity and serves as an organizer of the meristem (Scheres *et al.*, 1994). The root meristem can be further subdivided into a proximal (relative to the QC) and distal meristem, both consisting of cells with mitotic activity. Maintenance of the RAM requires a tight temporal and spatial regulation balancing production and differentiation of meristematic cells (Morrison and Spradling, 2008; Sozzani and Iyer-Pascuzzi, 2014).

The maintenance of the root stem cell niche is assured by transcription factors acting in several parallel pathways. The homeodomain transcription factor WUSCHEL-RELATED HOMEOBOX5 (WOX5) is expressed in the QC and serves to maintain QC and adjacent stem cells (Sarkar *et al.*, 2007). The GRAS family transcription factors SCARECROW (SCR) and SHORTROOT (SHR) (Sabatini *et al.*, 2003) are required for QC establishment and act together in specification of the endodermis, and members of the PLETHORA (PLT) family of AP2-type transcription factors respond to the auxin gradient in the root to control the size of the root meristem (Blilou *et al.*, 2005; Galinha *et al.*, 2007). During postembryonic development, root growth is directed by differential distribution of auxin and the establishment of an auxin concentration maximum at the root tip. Auxin transport is carried out by the influx carriers AUXIN RESISTANT1/LIKE AUX1 (AUX1/LAX), efflux transporters of the PINFORMED (PIN) family, and ABCB/P-GLYCOPROTEIN (PGP) efflux transporters (Benková *et al.*, 2003; Blilou *et al.*, 2005; Vieten *et al.*, 2007; Petrášek and Friml, 2009). Interestingly, auxin itself is a key regulator of its own transport by affecting the expression of its carriers (Vieten *et al.*, 2007; Petrášek and Friml, 2009).

At the cellular level, direct interpretation of differential auxin concentrations requires the action of the TRANSPORT INHIBITOR RESPONSE1/AUXIN SIGNALING F-BOX PROTEINS (TIR1/AFB) family. The *TIR1*/*AFB* genes encode F-Box proteins that are part of several SCF^TIR1/AFB^ E3 ubiquitin ligase complexes (Skp1·cdc53/cullin·F-box^TIR1/AFB^) (Dharmasiri *et al.*, 2005; Parry *et al.*, 2009). It has been shown that the presence of auxin in the binding pocket of TIR1 functions as a ‘molecular glue’ stabilizing the interaction with AUXIN/INDOLE-3-ACETIC ACID (AUX/IAA) co-repressor proteins. Once bound to the SCF^TIR1/AFB^ complexes, AUX/IAAs are ubiquitinated and subsequently degraded by the 26S proteasome (Gray *et al.*, 2001; Dharmasiri *et al.*, 2005; Kepinski and Leyser, 2005; Tan *et al.*, 2007). The turnover of AUX/IAA co-repressors, which interact with DNA-binding ARF proteins, allows the ARFs to exert their function as transcriptional regulators that control the expression of their target genes (Ulmasov *et al.*, 1997, 1999; Liscum and Reed, 2002; Tiwari *et al.*, 2004).

Single mutations in members of the TIR/AFB gene family cause a mild auxin-related phenotype, except for the *tir1-1* mutant, which is resistant to auxin and displays a shorter root than wild-type plants (Ruegger *et al.*, 1998). Higher order mutants of the TIR/AFB gene family members exhibit severe growth defects and increased auxin resistance (Dharmasiri *et al.*, 2005, Parry *et al.*, 2009). The *TIR1*/*AFB* genes are broadly expressed during various stages of plant development and their transcripts are present in overlapping domains, within embryos, seedling roots, emerging lateral roots, vascular bundles in cotyledons and mature leaves, and in mature floral organs (Dharmasiri *et al.*, 2005, Parry *et al.*, 2009). Regardless of their overlapping expression pattern, detailed studies revealed that the TIR1/AFB proteins have distinct biochemical and biological activities, and that TIR1 and AFB2 are the dominant auxin receptors controlling Arabidopsis root development (Parry *et al.*, 2009). Expression of the *TIR1*/*AFB* genes is regulated at multiple levels, and they are also subject to post-transcriptional control through microRNA *miR393* that negatively regulates *TIR1*, *AFB2*, and *AFB3* expression in response to pathogen attack (Navarro *et al.*, 2006; Parry *et al.*, 2009). Supporting the biotic stress-dependent regulation of the *TIR1*/*AFB* genes it was further shown that *miR393* overexpression results in auxin-resistant root growth and that expression of *miR393a* and *miR393b* is complementary to that of *pTIR1*:*TIR1-GUS*, consistent with *miR393* negatively regulating *TIR1* expression (Parry *et al.*, 2009). However, *miR393* does not contribute to the developmental regulation of the *TIR1*/*AFB* genes, as introduction of a mutation into the miR393 target sequence of *TIR1*, *AFB2*, and *AFB3* does not affect their expression under normal growth conditions (Parry *et al.*, 2009). The precise mechanisms that control differential expression of *TIR1*/*AFB* genes in different plant tissues are not yet known.

JLO, an LBD family transcription factor, plays an important role in plant development (Borghi *et al.*, 2007). Loss-of-function *jlo* mutants result in embryonic or seedling lethality. *JLO* is expressed in embryos, the root meristem and later at the boundaries between organ primordia and the remainder of shoot and organ meristems. *JLO* misexpression drastically affects organ initiation, leaf development and meristem maintenance (Borghi *et al.*, 2007, Rast and Simon, 2012). Some of these effects could be assigned to the misexpression of *KNOX* genes in incipient organ primordia, indicating that JLO can regulate meristematic gene functions. JLO shares this function with other LBD proteins, such as ASYMMETRIC LEAVES 2 (AS2). Importantly, JLO and AS2 physically interact in yeast and in planta (Rast and Simon, 2012). Furthermore, JLO can indirectly interact also with ASYMMETRIC LEAVES 1 (AS1) in the presence of AS2, suggesting that at least heterotrimeric complexes of these transcription factors exist in plants (Rast and Simon, 2012). *JLO* has been shown to be involved in numerous auxin-dependent developmental processes, such as embryonic development, organ primordia initiation and growth, and differentiation of vascular precursors (Borghi *et al.*, 2007; Soyano *et al.*, 2008; Rast and Simon, 2012). The strong patterning defects observed in *jlo* mutants were previously shown to be due to misregulation of the *BODENLOS*/*MONOPTEROS* (*BDL*/*MP*) pathway, and a resulting failure in auxin signaling. One consequence of this is the severely reduced expression of *PIN* and *PLT* family members in *jlo-2* mutant roots (Bureau and Simon, 2008).

Here we now show that impaired JLO function results in the stabilization of BDL and, as a consequence, in a misexpression of a number of auxin-regulated genes in addition to a failure to express auxin receptors at normal levels. Both the lack of expression of auxin receptor encoding genes and the resulting stabilization of AUX/IAA proteins in *jlo* mutants is causal for many of the developmental defects that we observe in *jlo* mutants. Furthermore, we find that JLO is not only necessary, but also sufficient for the expression of TIR1 and AFB1. Our findings disclose a new regulatory layer to the hierarchy of auxin signaling at the level of auxin perception.

## Materials and methods

### Plant material and growth conditions

The *jlo-2* (L*er*, Bureau *et al.*, 2010), *plt1-4* (L*er*, Aida *et al.*, 2004), *plt2-2* (L*er*, Aida *et al.*, 2004), and *tir1-1* (Col, Ruegger *et al.*, 1998) mutants were obtained from the Nottingham Arabidopsis Stock Centre (NASC). The *jlo-3* (*pst17018*), *jlo-4* (*pst19766*), *jlo-5* (*pst20504*), *jlo-6* (*pst00432*) and *jlo-7* (*pst13957*) mutations are in the Nossen (No-0) background and belong to the RIKEN collection (Rast and Simon, 2012). The origins of marker and other transgenic lines are as follows: *DR5rev::GFP* (Friml *et al.*, 2003), *DII::VENUS* (Brunoud *et al.*, 2012); *AUX1::AUX1-YFP* (Swarup *et al.*, 2004), *WOX5::NLS-GFP* (Nodine *et al.*, 2007), *PLT3::CFP* (Galinha *et al.*, 2007), *BDL::BDL-GUS* (Dharmasiri *et al.*, 2005), *TIR1::GUS*, *TIR1::TIR1-GUS*, *AFB1::GUS*, *AFB1::AFB1-GUS* (Parry *et al.*, 2009), *MP::MP-GFP* (Cole *et al.*, 2009), *SCR::SCR-YFP* (Heidstra *et al.*, 2004), *TIR1::TIR1-VENUS* (Wang *et al.*, 2016), *AFB1::AFB1-VENUS* (Stefan Kepinski), and *LexA35S::JLO-FLAG* (=*i35S::JLO-FLAG*) (Bureau *et al.*, 2010).

The following mutant lines were generated by crossing the strains: *plt1-4;plt2-2*, *jlo-2/+;plt1-4*, *jlo-2/+;plt2-2*, *jlo-2/+;plt1-4;plt2-2*, *jlo-2/+;tir1-1*, *DR5rev::GFP; jlo-2/+*, *DII-VENUS;jlo-2/+*, *AUX1::AUX1-YFP;jlo-2/+*, *WOX5::NLS-GFP;jlo-2/+*, *PLT3::CFP;jlo-2/+*, *BDL::BDL-GUS;jlo-2/+*, *TIR1::GUS;jlo-2/+*, *TIR1::GUS;jlo-5*, *TIR1::GUS;jlo-7*, *TIR1::TIR1-GUS;jlo-2/+*, *TIR1::TIR1-GUS;jlo-5*, *TIR1::TIR1-GUS;jlo-7*, *AFB1::GUS;jlo-2/+*, *AFB1::GUS;jlo-5*, *AFB1::GUS;jlo-7*, *AFB1::AFB1-GUS;jlo-2/+*, *AFB1::AFB1-GUS;jlo-5*, *AFB1::AFB1-GUS;jlo-7*, *MP::MP-GFP;jlo-2/+*, *SCR::SCR-YFP;jlo-2/+*, *TIR1::TIR1-VENUS;jlo-2/+*, and *AFB1::AFB1-VENUS;jlo-2/+*.

For JLO misexpression experiments, a *i35S::JLO-FLAG* line (Col) was crossed into *TIR1::GUS*, *TIR1::TIR1-GUS*, *AFB1::GUS*, *AFB1::AFB1-GUS*, *TIR1::TIR1-VENUS* and *AFB1::AFB1-VENUS* marker lines.

*Arabidopsis thaliana* seeds were surface fume sterilized in a sealed container with 100 ml bleach (chlorine gas) supplemented by 3 ml of 37% HCl for 3 h, then suspended in 0.1% agarose, and plated on a growth medium consisting of half-strength Murashige Skoog salts (Duchefa), 1% sucrose, 0.8% plant agar, MES (pH 5.8), stratified for 2 days in a 4°C dark room, and grown vertically in a growth chamber under constant light conditions at 16 or 21°C. For auxin treatment, seedlings were mounted in liquid MS medium, containing 20 µM of indole-3-acetic acid (IAA) or 10 µM of 2,4-dichlorophenoxyacetic acid (2,4-D). To inhibit protein degradation by proteasome, proteasome inhibitor *N*-benzyloxycarbonyl-L-leucyl-L-leucyl-L-leucinal (MG132) was used, and seedlings were pretreated with 50 μM MG132 for 1 h, followed by 1 h incubation in 20 μM IAA.

*Nicotiana benthamiana* plants were grown for 4 weeks in a greenhouse under controlled conditions. Induction of transgene expression was performed by spraying with 20 μM β-estradiol and 0.1% Tween 20.

### Binary constructs and plant transformation

For the analysis of the JLO expression pattern, a *JLO::GFP* line was constructed. For this, the *JLO* promoter region (3273 bp upstream of the ATG) was synthesized (Life Technologies), introduced into pDONRZeo, and recombined into pMDC161 (Curtis and Grossniklaus, 2003). Subsequent transformation of *Arabidopsis thaliana* Columbia plants was carried out with the floral dip method (Clough and Bent, 1998). Transgenic plants were selected on MS medium containing hygromycin (15 mg ml^–1^).

The AFB1::AFB1:VENUS reporter was constructed by PCR amplification of a 3 kb promoter region and the AFB1 coding sequence, including all introns, and in frame fusion of the last exon to the VENUS sequences, followed by 2 kb of genomic sequences from the 3′ region of the gene. This fragment was cloned into the pGREEN 0229 backbone and transformed into the *afb1-3* mutant background. Primers used were *Eco*R1/pAFB1F (5′-TCAGAATTCATGGAGAACATAAACGAATCAA CTATAGTC-3′), AFB1/*Bam*H1 (5-CTAGGATCCCTTTAT GGCTAGATGTGAAACTCCATTC-3′), *Bam*H1/Venus (5′-CTAGGATCCGTGAGCAAGGG CGAGGAGCT-3′), VenusStop/*Not*1 (5′-ATAGCGGCCGCTAC TTGTACAGCTCGTCCATGCCGAGA-3′), *Not*1-3′-AFB1 (5′-TATGCGGCCGCACTTGCTGCTTCAGTCATATTTT CCTTTCC-3′) and 3′-AFB1/*Not*1 (5′-TTAGCGGCCGCATG TGATTATTGACTATGTTTACCCTGC-3′).

For *2x35S::TIR1-FLAG* and *2x35S::AFB1-FLAG* transgene construction, the *TIR1* (AT3g62980) and *AFB1* (At4g03190) coding region without stop codons were amplified from Col-0 genomic DNA with the primers TIR1 fwd (5′-ATGCAGAAGCGAA TAGCCTTGTCGT-3′), TIR1 rev (5′-TTATAATCCGTTA GTAGTAATGATT-3′), AFB1 fwd (5′-ATGGGTCTCCGA TTCCCACCTAAGG-3′), and AFB1 rev (5′-TTACTTTATGGCTAG ATGTGAAACT-3′). The C-terminal FLAG tag (GCCTCGTCAGTGATAAAACGAGAAGACTACAA) and the *attB* recombination sites were added via PCR-mediated ligation. According to the manufacturer’s instructions (Gateway manual; Invitrogen) the PCR fragment was recombined into pDONR221 and finally into the binary plant transformation vector pMDC32 (Curtis and Grossniklaus, 2003). Subsequent transformation of *Arabidopsis thaliana jlo-2*/+ plants was carried out with the floral dip method (Clough and Bent, 1998). Transgenic plants were selected on MS medium containing hygromycin (15 mg ml^–1^) and kanamycin (25 mg ml^–1^).

For protein interaction studies, *attB* sites were added via PCR-mediated ligation to coding regions of *JLO*, *BDL*, or *MP.* PCR products were introduced into pDONR201 and eventually recombined into pABindGFP, pABindCherry, or pABindFRET (Bleckmann *et al.*, 2010). Binary vectors were transformed in *Agrobacterium tumefaciens* GV3101 pMP90 (Koncz *et al.*, 1984) according to the manufacturer’s instructions (Invitrogen). Abaxial leaf sides of *Nicotiana benthamiana* plants were infiltrated as described in Bleckmann *et al.* (2010). Transgene expression was induced 48 h after infiltration by spraying with 20 µM β-estradiol, 0.1% Tween 20 and analysed within 12 h after induction.

### *E*_FRET_ measurements via acceptor photobleaching

*Nicotiana benthamiana* leaf epidermal cells were examined with a ×40, 1.3 numerical aperture Zeiss oil-immersion objective using a Zeiss LSM 510 Meta confocal microscope. Förster resonance energy transfer efficiency (*E*_FRET_) was measured via green fluorescent protein (GFP) fluorescence intensity increase after photobleaching of the acceptor mCherry (Bleckmann *et al.*, 2010). The percentage change of the GFP intensity directly before and after bleaching was analysed as *E*_FRET_=(GFP_after_–GFP_before_)/GFP_after_×100. All photobleaching experiments were performed in the nucleus. A minimum of 25 measurements were performed for each experiment. Significance was analysed using Student’s *t*-test.

### Gene expression analysis

Reporter gene analysis was performed in the F3 generation after genetic crossing. To detect β-glucuronidase (GUS) activity, 5-day-old seedlings were incubated in reaction buffer containing 0.1M sodium phosphate buffer (pH 7), 1mM ferricyanide, 1mM ferrocyanide, 0.1% Triton X-100 and 1mgml^−1^ X-Gluc for 10 min to 8h in dark at 37°C. Afterwards, chlorophyll was removed by destaining in 70% ethanol and seedlings were cleared with 70% (w/v) chloral hydrate–10% (v/v) glycerol solution. Analysis of fluorescence reporter expression was performed using a Zeiss LSM780 confocal microscope. Counterstaining of root cell walls was achieved by mounting roots in 10 µM propidium iodide (PI).

The RNeasy Plant Mini Kit (Qiagen) was used for RNA extraction from roots. RNA was treated with DNase (Fermentas) and transcribed into cDNA using SuperScriptII (Invitrogen). Quantitative reverse transcription PCR (qRT-PCR) was performed in triplicates using the Mesa Blue Sybr Mix (Eurogentec) and a Chromo4 real-time PCR machine (Bio-Rad). Oligonucleotide sequences are given in Supplementary Table S1 at *JXB* online. Expression levels were normalized to the reference gene *At4g34270* (Czechowski *et al.* 2005). The *JLO* misexpression experiments were performed as described in (Rast and Simon, 2012).

### Phenotypic analysis and microscopy

Root architecture was studied with the modified pseudo-Schiff propidium iodide (mPSPI) method (Truernit *et al.*, 2008) and imaged with a Zeiss LSM 780 laser scanning microscope. Image acquisition was carried out with an Axiocam HR camera attached to a Zeiss Axioscope II microscope. For root length analysis, seedlings were photographed and root lengths were measured with ImageJ (http://rsb.info.nih.gov/ij).

Scanning electron microscopy (SEM) analysis of SAMs was performed as described previously (Kwiatkowska, 2004). Briefly, the images showing the surface of individual shoot apices were obtained using replicas (dental polymer molds) taken from the surface of individual shoot apices. Epoxy resin casts prepared from these molds were sputter-coated and were observed by scanning electron microscope (LEO435VP). Images were processed in ImageJ software and assembled in Adobe Illustrator.

### Chromatin immunoprecipitation assay

We took advantage of a previously described Arabidopsis inducible overexpression line, *i35S::JLO-FLAG*, that carries an estradiol-inducible *JLO-FLAG* transgene (Bureau *et al.*, 2010). A monoclonal antibody directed against the FLAG epitope of the JLO–FLAG fusion protein was used in a chromatin immunoprecipitation (ChIP) experiment, which was performed as described in Schubert *et al.* (2006) using 1 g of tissue seedlings at 5 days after germination (DAG) and monoclonal ANTI-FLAG^®^ M2 antibodies (Sigma-Aldrich). Inducible production of the JLO–FLAG fusion protein was previously confirmed by Western blotting with an anti-FLAG-antibody (Bureau *et al.*, 2010). Nuclear extracts derived from *i35S::JLO-FLAG* seedlings aged 5 DAG at 24 h after induction of JLO–FLAG expression were sonicated to obtain DNA fragments ranging from 250 to 500 bp. After immunoprecipitation, the enrichment of the TIR1 and AFB1 promoters was estimated by qPCR to define the regions of the TIR1 and AFB1 promoter showing enrichments of percentage input yield compared with adjacent promoter regions and with wild-type control. Input and immunoprecipitation DNA was diluted 1:10, and 2 μl was used for real-time PCR. The SYBR Green II Master kit was used for all qPCRs, and ACTIN2 was utilized as negative controls. Oligonucleotides were designed in the program Prime3 (http://www.bioinformatics.nl/cgi-bin/primer3plus/primer3plus.cgi, for oligonucleotides, see Supplementary Table S2). To analyse the ChIP enrichment from qPCR data, the percentage input method was used. ChIP data were obtained from single experiments, but similar data were acquired from three independent experiments. Values for immunoprecipitation were referenced to input values.

Six primer sets for *TIR1* and three for *AFB1* were used to estimate the relative enrichment of their promoters and other primer pairs for introns and for coding regions of respective genes. Primers were designed to amplify regions of a promoter of approximately 200 bp and cover the whole promoter sequence located 2446 bp upstream of the transcriptional start site (TSS) for TIR1 and 1238 bp upstream of the TSS for AFB1 (see Supplementary Fig. S1).

## Results

### Establishment and maintenance of the root stem cell niche depends on JLO

**Fig. 1.**
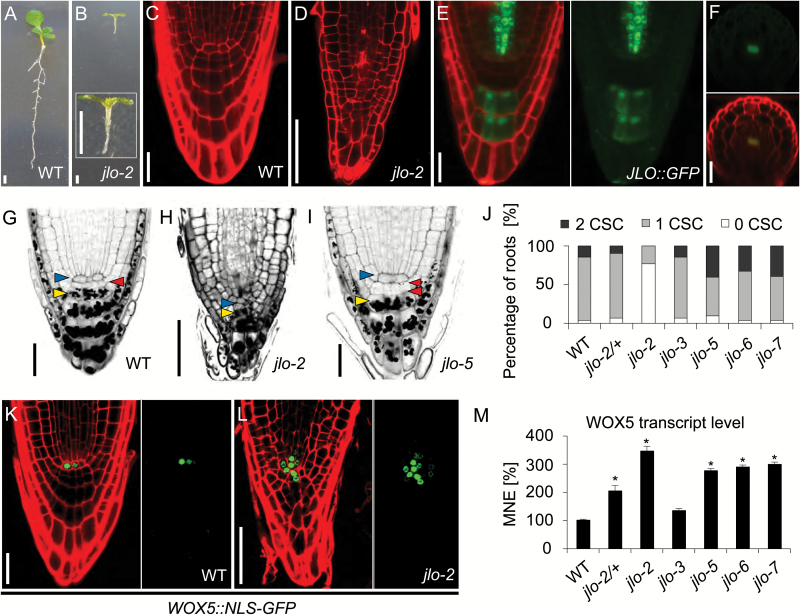
Phenotype of *jlo* mutants and *JLO::GFP* expression. (A, B) Phenotype of wild-type (A) and *jlo-2* mutant seedlings (B). The inset in (B) shows a higher magnification of a *jlo-2* mutant seedling. (C, D) Root tip of wild-type (C) and *jlo-2* mutant seedlings (D), stained with propidium iodide (PI; red signal on cell walls). (E, F) Expression of *JLO::GFP* reporter in the root tip (E) and in a root cross-section at approximately 250 μm from the tip (F), PI stained, green signal from GFP. (G–I) Differentiation status of distal root meristems of wild-type (G), *jlo-2* mutant (H), and *jlo-5* mutant (I), mPSPI staining of cell walls and starch granules, columella stem cells (red arrowheads), quiescent center (blue arrowheads), and columella cells (yellow arrowheads). (J) Quantification of columella stem cell (CSC) number as percentage of wild-type, *jlo-2/+*, *jlo-2*, *jlo-3*, *jlo-5*, *jlo-6*, and *jlo-7* mutant seedlings. (K, L) Expression of *WOX5::NLS-GFP* reporter in the root tip of wild-type (k) and *jlo-2* mutant seedlings (L), stained with PI. (M) *WOX5* transcript levels were analysed by qRT-PCR in roots of wild-type, *jlo-2/+*, *jlo-2*, *jlo-3*, *jlo-5*, *jlo-6*, and *jlo-7* seedlings. All seedlings were analysed at 5 days after germination. CSC, columella stem cell; MNE, mean normalized expression; WT, wild-type. Asterisks mark a significant difference from wild-type (*P≤0.01, analysed by Student’s *t*-test). Scale bars: 50 µm. Error bars in (M) indicate standard error.

JAGGED LATERAL ORGANS (JLO), a transcription factor from the LATERAL ORGAN BOUNDARY DOMAIN (LBD) family, was shown to be required for precise pattern formation from embryogenesis onwards, as loss-of-function *jlo* mutants arrest embryogenesis at the globular stage or display seedling lethality (Borghi *et al.*, 2007; Bureau *et al.*, 2010). The roots of the strong *jlo-2* mutants remain short and, from the fifth day after germination (DAG) onwards cease to develop further (Bureau *et al.*, 2010, Fig. 1A, B). In contrast to wild-type roots, the meristem of *jlo-2* mutants appears disorganized and a structurally distinct QC with surrounding stem cells cannot be readily identified (Fig. 1C, D, G, H, J) (Bureau *et al.*, 2010). This severe disruption of development in *jlo-2* mutants confounds functional analysis of later developmental stages, and we therefore analysed a series of phenotypically milder *jlo* alleles (*jlo-3* to *jlo-7*) that retain some residual JLO function (Rast and Simon, 2012). Distal to the QC, wild-type roots maintain a single layer of columella stem cells (CSCs) that gives rise to four to five layers of differentiated columella cells (CCs), which contain starch granules (Fig. 1G and Supplementary Fig. S2). The CSC layer appears as two cell tiers following the immediate division of the CSCs. Analysis of homozygous *jlo-3* and *jlo-5* to *jlo-7*, which are weak alleles of *jlo* with residual function, revealed that an increased percentage of *jlo-5*, *jlo-6* and *jlo-7* roots carried two CSC layers (*n*≥50 for each, *P*<0,001); *jlo-3* mutants were like wild-type, while *jlo-2* mutants maintained one or no CSCs (Fig. 1H–J and Supplementary Fig. S2A–D). This suggests that the differentiation of CSC daughter cells into columella cells in *jlo-5* to *jlo-7* mutants is delayed (where JLO activity is reduced), but precocious in *jlo-2* (where JLO activity is almost absent). In wild-type, CSCs are maintained by the homeodomain transcription factor WOX5, which is in turn regulated by *CDF4* (Pi *et al.*, 2015) and by auxin (Sarkar *et al.*, 2007; Ding and Friml, 2010; Tian *et al.*, 2014). *WOX5* is expressed in the QC and controls the number of CSC layers in a dosage-dependent manner (Sarkar *et al.*, 2007; Ding and Friml, 2010). We therefore asked if *WOX5* expression was affected by the presence of *JLO*. In contrast to wild-type, *WOX5* expression in *jlo-2* mutant roots expands from the QC into the adjacent stem cells (Fig. 1K, L) and we found increased *WOX5* transcript levels in roots of the *jlo-2* and *jlo-5* to *jlo-7* mutants, but not in *jlo-3* (Fig. 1M). Thus, *JLO* represses *WOX5* expression outside of the QC in wild-type; however, the differential response of CSCs to altered JLO dosage cannot be simply explained through regulation of *WOX5* alone. We therefore studied the expression pattern of *JLO* in more detail to investigate if *JLO* could regulate *WOX5* in the QC or surrounding cells. A transgenic *JLO* reporter line (*pJLO::GFP*) showed expression in the root vascular bundle, commencing immediately proximal to the quiescent center (QC) in the vascular initials (Fig. 1E, F). At approximately 250 μm from the root tip GFP signal was discernible in the metaxylem cells (Fig. 1F). In addition, *JLO* was highly expressed in the second and third CC layer (Fig. 1E). Thus, the expression patterns of *JLO* and *WOX5* do not overlap, but rather appear mutually exclusive, suggesting that *JLO* could serve to repress *WOX5* outside of the QC during normal development.

### JLO acts with the auxin/PLETHORA pathway in root meristem maintenance

**Fig. 2.**
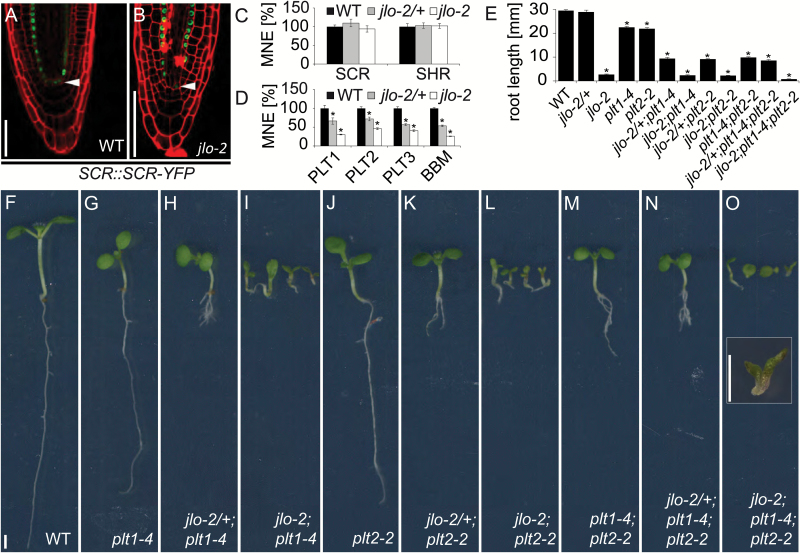
Genetic interaction between *JLO*, *SCR*, and *PLT.* (A, B) Expression of an S*CR::SCR-YFP* reporter in wild-type (A) and *jlo-2* (B) mutant roots, stained with PI. Arrowheads mark the QC. (C, D) qRT-PCR analysis of SCR, SHR expression (C) and PLT/BBM (D) expression in roots of wild-type, *jlo-2/+*, and *jlo-2*. (E–O) Root length of the indicated seedling genotypes. Quantitative measurements of root length (E), representation of the wild-type (F), *plt1-4* (G), *jlo-2/+;plt1-4* (H), *jlo-2;plt1-4* (I), *plt2-2* (J), *jlo-2/+;plt2-2* (K), *jlo-2;plt2-2* (L), *plt1-4;plt2-2* (M), *jlo-2/+;plt1-4;plt2-2* (N), and *jlo-2;plt1-4;plt2-2* (O) mutant seedlings. The inset in (O) shows a higher magnification of a *jlo-2;plt1-4;plt2-2* triple mutant seedling. All seedlings were analysed at 5 days after germination. MNE, mean normalized expression; WT, wild-type. Scale bars: 50 µm in (A–E) and 2 mm in (F–O). Error bars in (C–E) indicate standard error. Asterisks mark a significant difference from wild-type (*P≤0.01, analysed by Student’s *t*-test).

QC specification and *WOX5* expression require the activity of two GRAS family transcription factors, SCARECROW (SCR) and SHORTROOT (SHR) (Sabatini *et al.*, 2003; Sarkar *et al.*, 2007). Mutations in either gene result in a premature differentiation of root meristem cells and loss of QC function. We asked if JLO function is mediated by either SHR or SCR. Therefore, we monitored *SCR* expression using a *SCR::SCR-YFP* reporter construct. In wild-type, SCR–YFP signals are detectable in the root endodermis, the endodermis/cortex initial and the QC (Fig. 2A). Roots of homozygous *jlo-2* mutants showed *SCR* expression in cells of the presumptive QC, as well as in a single cell layer directly adjacent to the vascular tissues, which thus likely has endodermis identity (Fig. 2B). Using quantitative reverse transcription PCR (qRT-PCR), we then showed that neither *SCR* nor *SHR* expression levels, which mutually promote the expression of each other, are altered in *jlo-2* mutant roots (Fig. 2C), indicating that *JLO* regulates *WOX5* expression and root meristem maintenance independent of the *SCR/SHR* pathway.

Another pathway promoting QC identity and root meristem maintenance comprises members of the PLETHORA (PLT) family (also called AINTEGUMENTA-LIKE) of AP2-type transcription factors (Blilou *et al.*, 2005) and is auxin dependent. We previously found that genes belonging to the *PLT* family (*PLT1*, *PLT2*, and *BABYBOOM* (*BBM*)/*PLT4*) are expressed at lower levels in *jlo-2* roots, and that *PLT1*, *2*, and *4* are transcriptionally up-regulated upon inducible *JLO* overexpression (Bureau *et al.*, 2010). In wild-type, *PLT3* is highly expressed in the stem cell niche and the CCs at the basal root tip, and expressed in a gradient in epidermal and vascular tissues (see Supplementary Fig. S3A). In a *jlo-2* mutant background, PLT3 is expressed in a similar pattern but at lower levels (Supplementary Fig. S3B). Importantly, qRT-PCR analysis with RNA prepared from whole seedlings at 5 DAG showed a down-regulation of all four *PLT* genes in *jlo-2* mutants, and even showed a reduction in expression of all genes in *jlo-2/+* heterozygous seedlings by approximately 40% (Fig. 2D). Thus, the expression of all four *PLT* genes tested here is highly sensitive to the dosage of *JLO* throughout the plant.

The *PLT* genes contribute redundantly and in a dosage-dependent manner to root growth and RAM maintenance. Therefore, single mutants display only mild phenotypes while roots of double or triple mutants show strong patterning defects (Aida *et al.*, 2004; Galinha *et al.*, 2007), and the RAM disorganization of various *plt*/*bbm* allelic combinations partially resembles that observed in *jlo-2* mutants. Thus, reduction of PLT activity could be causal for the developmental defects of *jlo* mutants. To further disclose the genetic relationship between *JLO* and the *PLT* genes, we created double and multiple mutant combinations of *jlo-2/*+ with the *plt1-4* and *plt2-2* loss-of-function alleles (see Supplementary Table S3). The F3 progeny of *jlo-2/+;plt1-4*, *jlo-2/+;plt2-2*, and *jlo-2/+;plt1-4;plt2-2* plants were examined at 5 DAG. Compared with plants homozygous for mutations in individual *PLT* genes, root length and meristem organization were stepwise further reduced when the *plt* mutants were combined with *jlo-2* heterozygous and homozygous plants (Fig. 2E–O, Supplementary Fig. S3C–J). Thus, in all mutant combinations analysed, we found that *jlo* mutants enhanced all phenotypes of *plt* mutants. Since *PLT* genes act redundantly, this could indicate that *JLO* acts fully through the *PLT* pathway. However, *JLO* may also affect the regulation of other, *PLT*-independent processes and target genes.

### JLO regulates the earliest steps of auxin signaling

The fact that impaired JLO function affects the expression of *PLT* family members but not *SHR* or *SCR* genes, together with *JLO*-dependent restriction of *WOX5* expression, could suggest that *JLO* acts specifically in an auxin-dependent pathway to control root meristem development. This assumption is consistent with the misexpression of a number of auxin-regulated target genes in *jlo* mutant backgrounds. These genes not only include members of the PIN family of auxin efflux carrier, as previously shown (Bureau *et al.*, 2010, Rast and Simon, 2012), but also AUX1, a member of the AUX/LAX family of auxin influx carriers that act to stabilize the auxin gradient (Marchant *et al.* 2002; Bainbridge *et al.*, 2008). Monitoring the expression of an *AUX1::AUX1-YFP* reporter gene in *jlo-2* mutant background revealed an essentially unaltered expression pattern compared with wild-type, but an overall reduction in expression level (see Supplementary Fig. S4A, B). Thus, JLO function is essential to facilitate both active auxin export and auxin import. To further reveal whether *JLO* regulates auxin-dependent gene expression or auxin signaling, we assessed the overall auxin response in *jlo-2* mutants. The transcriptional response to auxin can be monitored using the *DR5rev::GFP* reporter, which consists of several ARF-binding sites driving the expression of GFP (Ulmasov *et al.*, 1997; Sabatini *et al.*, 1999; Benková *et al.*, 2003; Heisler *et al.*, 2005), while the DII-VENUS sensor (Brunoud *et al.*, 2012) allows the monitoring of local degradation of AUX/IAAs depending on auxin levels and perception.

**Fig. 3.**
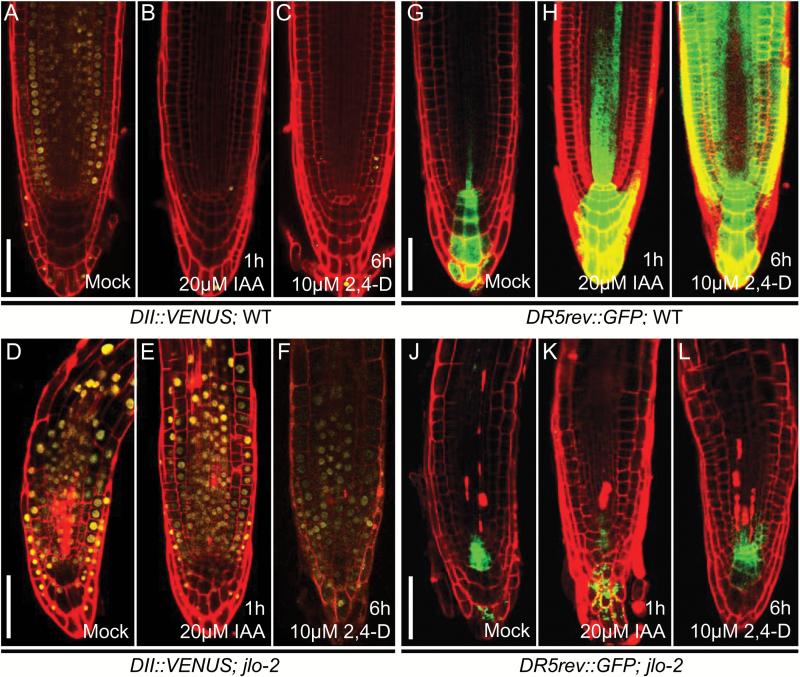
*JLO* is required for the response to auxin. (A–C) Expression of *DII-VENUS* in untreated wild-type roots (A), in wild-type roots treated with auxin (IAA) for 1 h (B) and in wild-type roots treated with synthetic auxin (2,4-D) for 6 h (C). (D–F) Expression of *DII-VENUS* in untreated *jlo-2* roots (D), in *jlo-2* roots treated with auxin (IAA) for 1 h (E), and in *jlo-2* roots treated with synthetic auxin (2,4-D) for 6 h (F). (G–I) Expression of *DR5rev::GFP* in untreated wild-type roots (G), in wild-type roots treated with auxin (IAA) for 1 h (H), and in wild-type roots treated with synthetic auxin (2,4-D) for 6 h (I). (J–L) Expression of *DR5rev::GFP* in untreated *jlo-2* roots (J), in *jlo-2* roots treated with auxin (IAA) for 1 h (K) and in *jlo-2* roots treated with synthetic auxin (2,4-D) for 6 h (L). All seedlings were analysed at 5 days after germination. 2,4-D, 2,4-dichlorophenoxyacetic acid; IAA, indole-3-acetic acid; mock, untreated seedlings. Scale bars: 50 µm.

Auxin is transported through the root stele toward the basal root tip to generate a maximum at the QC, the surrounding initials and the CC. Consistent with this, we observed reduced DII-VENUS signals in the root stele and an absence of signals at the tip of wild-type roots (Fig. 3A) indicating a local, auxin-dependent degradation of the fusion protein. The *DR5rev:GFP* reporter appeared to be expressed in a mostly complementary pattern (Fig. 3G). Treatment of wild-type roots with natural auxin (IAA) or synthetic auxin analogues (2,4-D) resulted in a strong reduction of DII-VENUS signals (Fig. 3B, C) while the *DR5rev:GFP* expression was up-regulated (Fig. 3H, I). We then investigated DII-VENUS signals and *DR5rev:GFP* expression in homozygous *jlo-2* mutants. We found that DII-VENUS protein was expressed in the mutant roots (Fig. 3D), overlapping with a strongly reduced maximum of *DR5rev:GFP* expression (Fig. 3J). Moreover, the DII-VENUS protein abundance and *DR5rev:GFP* expression showed no clear response to artificially increased auxin contents in a *jlo-2* mutant background (Fig. 3E, F, K, L). These results indicate that *JLO* regulates both auxin-dependent gene expression and auxin signaling, as JLO function is required for proper auxin response in the root tip and additionally seems to be necessary to mediate AUX/IAA degradation.

### JLO mediates AUX/IAA degradation to allow auxin-induced gene expression

**Fig. 4.**
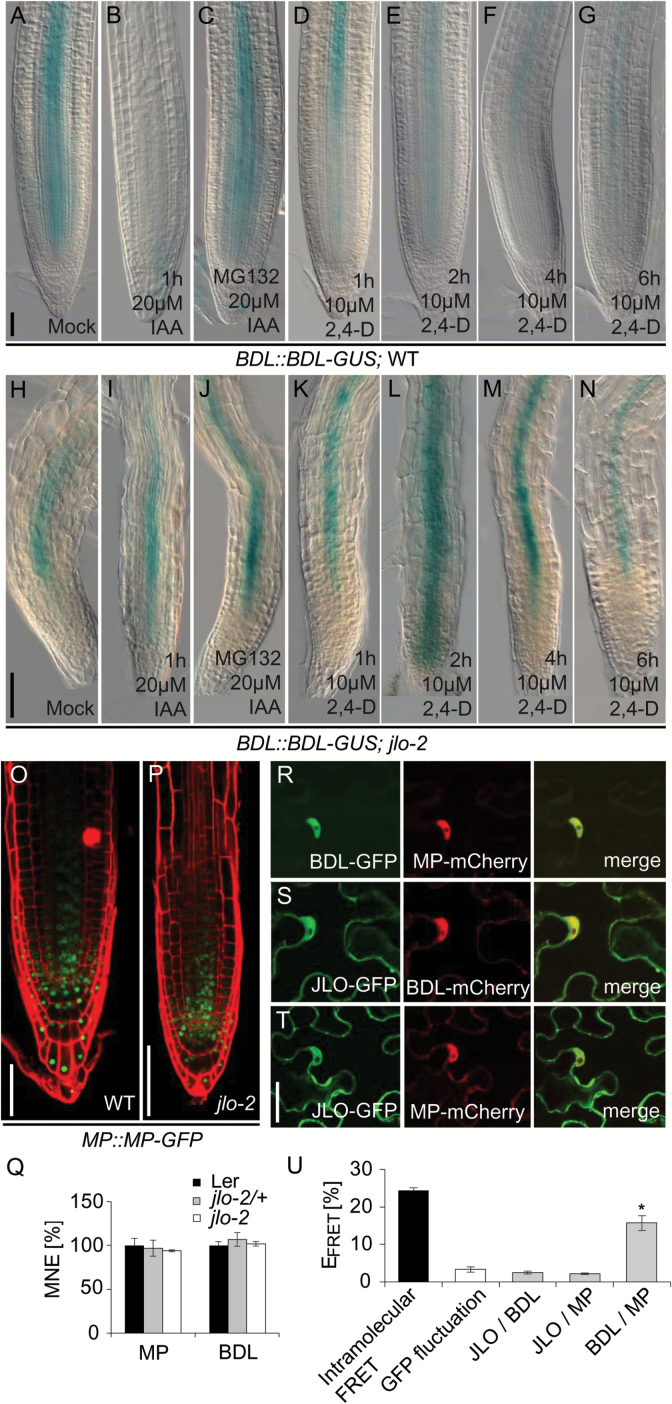
The AUX/IAA protein BODENLOS is stabilized in *jlo-2* mutants. (A–G) Expression of *BDL::BDL-GUS* in untreated wild-type roots (A), in wild-type roots treated with auxin (IAA) (B), MG132 and auxin (C), 2,4-D for 1 h (D), 2,4-D for 2 h (E), 2,4-D for 4 h (F), and 2,4-D for 6 h (G). (H–N) Expression of *BDL::BDL-GUS* in untreated *jlo-2* roots (H), *jlo-2* treated with auxin (IAA) (I), MG132 and auxin (J), 2,4-D for 1 h (K), 2,4-D for 2 h (L), 2,4-D for 4 h (M), and 2,4-D for 6 h (N). (O, P) Expression of an *MP::MP-GFP* reporter in the root tip of wild-type (O) and *jlo-2* (P). (Q) *MP* and *BDL* transcript levels analysed by qRT-PCR in roots of wild-type, *jlo-2/+*, and *jlo-2* seedlings. (R–U) FRET-based protein interaction analysis. Co-localization of fluorescent protein tagged BDL, MP, or JLO in *N. benthamiana* epidermis cells: (R) co-localization of BDL–GFP and MP–mCherry, (S) co-localization of JLO–GFP and BDL–mCherry, and (T) co-localization of JLO–GFP and MP–mCherry. (U) *E*_FRET_ measured after transient expression of FP-tagged protein in epidermis cells of *N. benthamiana*. Intramolecular *E*_FRET_ obtained by direct fusion of GFP to mCherry (black column) and GFP background fluctuation (white column) were calculated as positive and negative controls. All seedlings were analysed at 5 days after germination. 2,4-D, 2,4-dichlorophenoxyacetic acid; *E*_FRET_, FRET efficiency; IAA, indole-3-acetic acid; MG132, *N*-benzyloxycarbonyl-L-leucyl-L-leucyl-L-leucinal (proteasome inhibitor); MNE, mean normalized expression; mock, untreated seedlings; WT, wild-type. Asterisks mark a significant difference from controls (*P≤0.01, analysed by Student’s *t*-test). Scale bars: 50 µm for (A–P) and 10 µm for (R–T). Error bars in (Q) and (U) indicate standard error.

Previous genetic studies indicated that *JLO* acts in the *BDL/MP* pathway to regulate auxin-induced gene expression programs. However, the discrete hierarchy of the genes remained unclear (Bureau *et al.*, 2010). Interestingly, *mp* loss-of-function and *bdl* gain-of-function mutants lose the embryonic root and carry reduced hypocotyls and vascular systems (Hardtke and Berleth, 1998; Hamann *et al.*, 2002), similar to *jlo* loss-of-function mutants (Bureau *et al.*, 2010). This raises three possibilities: (1) JLO may be a transcriptional regulator of *MP* or *BDL* expression, (2) JLO may physically interact with the BDL and/or MP proteins, and (3) because the *bdl* gain-of-function mutation causes a stabilization of the BDL protein, JLO could be required for auxin-dependent BDL degradation (Hamann *et al.*, 2002). To distinguish between these possibilities, we first assayed *MP* and *BDL* expression in the *jlo-2* mutant background. We observed unaltered expression of an *MP::MP-GFP* and a *BDL::BDL-GUS* reporter in *jlo-2* mutant roots compared with wild-type (Bureau *et al.*, 2010; Fig. 4A, H, O, P). qRT-PCR analysis also confirmed no significant alterations in *MP* or *BDL* transcript levels in *jlo-2* mutants (Fig. 4Q). Thus, *JLO* does not regulate *MP* or *BDL* transcription.

To assay for protein interactions *in planta*, JLO, MP, and BDL were transiently expressed in *Nicotiana benthamiana* leaf epidermal cells as fusions to the fluorescent proteins GFP or mCherry. Interaction between proteins was then determined by measuring fluorescent donor dequenching after acceptor bleaching, which is an indicator of Förster resonance energy transfer (FRET) between interacting proteins. Apparent FRET efficiencies (*E*_FRET_) between the GFP and mCherry pairs were then calculated as the percentage increase of GFP fluorescence after photobleaching of mCherry (Albertazzi *et al.*, 2009; Bleckmann *et al.*, 2010). All fusion proteins were found to be localized in the cytoplasm and enriched in the nucleoplasm (Fig. 4R–T). Therefore, we performed all photobleaching experiments and *E*_FRET_ measurements in the nucleus. We found, consistent with previously published yeast GAL4 interaction studies, a clear MP/BDL (*E*_FRET_=15.7 ± 2.0; Weijers *et al.*, 2006) interaction in both reciprocal GFP–mCherry combinations (Fig. 4U). However, we could not detect a significant protein interaction between JLO and MP (*E*_FRET_=2.2 ± 0.2) or BDL (*E*_FRET_=2.5 ± 0.4) (Fig. 4U). Together, our results indicate that JLO neither regulates *MP* or *BDL* expression nor interacts directly with either of them at the protein level.

We next examined the possibility that impaired *JLO* function interferes with auxin-dependent BDL degradation. To this end we modified the auxin content in wild-type and *jlo-2* mutant roots that express a BDL–GUS fusion protein from its endogenous promoter. Consistent with previously published results (Dharmasiri *et al.*, 2005), we found that the BDL–GUS protein is destabilized by a 1 h treatment with 20 µm IAA in wild-type roots (100%; *n*=69; Fig. 4B), while mock treated controls showed a GUS staining in the root stele (100%; *n*=67; Fig. 4A). Additional application of the proteasome inhibitor MG132 confirmed that this auxin-dependent BDL degradation requires the proteasome. Wild-type roots that were pretreated with MG132, followed by incubation in 20 µM IAA for 1 h, displayed GUS signals comparable to the untreated controls (100%; *n*=32; Fig. 4C). In contrast, in 78% of the analysed *jlo-2* roots (*n*=139), GUS signals were still present after 1 h IAA treatment (Fig. 4I). Our results also showed that pretreatment with MG132 only slightly increased the number of GUS stained *jlo-2* roots after IAA application (82%; *n*=48; Fig. 4J). We then used the transport-independent auxin analogue 2,4-D in our GUS assay to exclude that the deficiency in BDL–GUS degradation is simply due to a failure to transport the exogenously applied auxin. Within a 1–6 h treatment, we found a drastic reduction in BDL–GUS signals in the wild-type control (*n*≥25 for each experiment; Fig. 4D–G), but not in homozygous *jlo-2* mutants (*n*≥25; Fig. 4K–N). Since loss of JLO function causes a stabilization of the BDL–GUS fusion protein, we concluded that JLO function is required to mediate AUX/IAA degradation to eventually allow ARF activity and regulation of auxin-induced gene expression programs.

### JLO mediates auxin perception through the *TIR1*/*AFB1*-signaling pathway

**Fig. 5.**
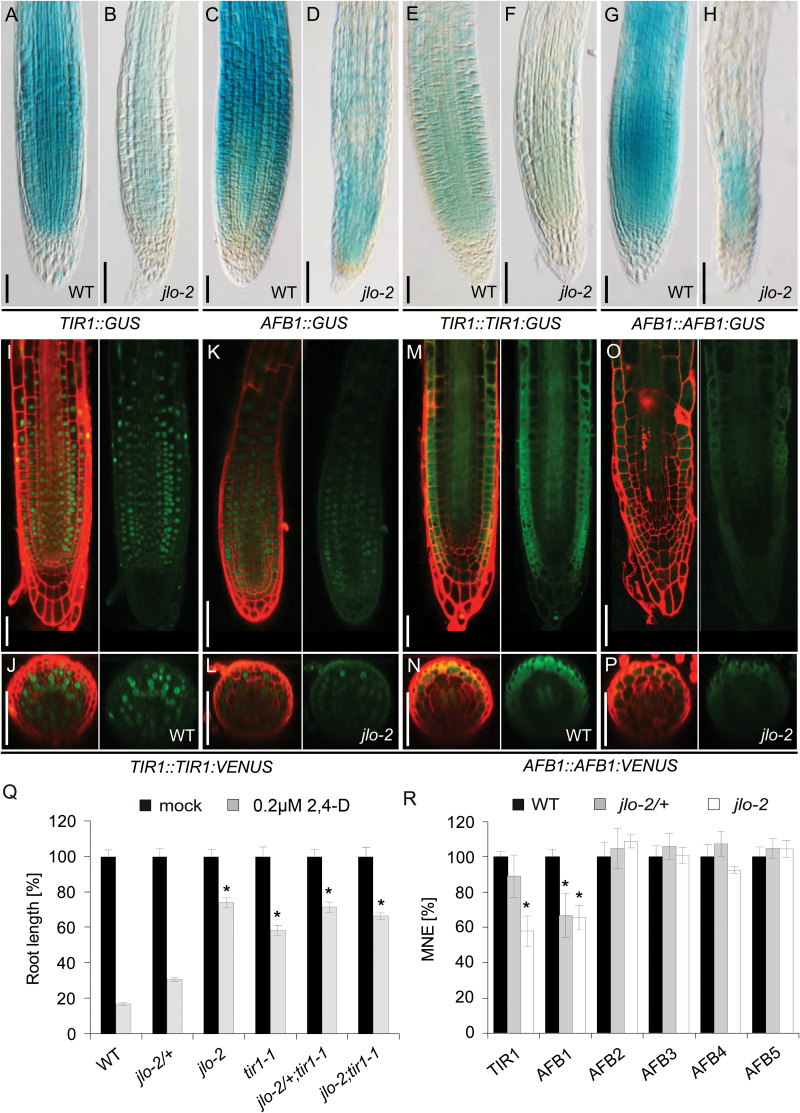
*JLO* controls expression of *TIR1* and *AFB1* in Arabidopsis roots. (A–H) Expression of *TIR1::GUS* (A, B), *AFB1::GUS* (C, D) transcriptional reporter, and *TIR1::TIR1:GUS* (E, F), *AFB1::AFB1:GUS* (G, H) translational reporter lines in the root tips of wild-type (A, C, E, G) or *jlo-2* (B, D, F, H). (I–P) Expression of *TIR1::TIR1:VENUS* (I–L) and *AFB1::AFB1:VENUS* (M–P) transcriptional reporter in the root tips of wild-type (I, J, M, N) or *jlo-2* (K, L, O, P). (J, L, N, P) Cross-sections at approximately 250 μm from the tip. Roots were stained with PI. (Q) Root length of plants treated with 0.2 µM 2,4-D, given as percentage of length of untreated seedlings of the same genotypes. (R) *TIR1* and *AFB1-5* transcript levels analysed by qRT-PCR in roots of wild-type, *jlo-2/+*, and *jlo-2* seedlings, given as percentage of wild-type levels. All seedlings were analysed at 5 days after germination. 2,4-D, 2,4-dichlorophenoxyacetic acid; MNE, mean normalized expression; mock, untreated seedlings; WT, wild-type. Asterisks mark a significant difference from wild-type (*P≤0.01, analysed by Student’s *t*-test). Scale bars: 50 µm. Bars in (Q, R) indicate standard error.

Our results provide evidence that JLO is required for AUX/IAA degradation in response to auxin. Members of the TIR1/AFB family of auxin receptors directly link auxin perception to the degradation of AUX/IAA proteins (Dharmasiri *et al.*, 2005; Kepinski and Leyser 2005). We used qRT-PCR assays to analyse if the expression levels of the six *TIR1*/*AFB* genes (*TIR1*, *AFB1*, to *AFB5*) is altered in the *jlo-2* mutant background. When RNA from whole seedlings at 5 DAG was analysed, we found a significant reduction of *TIR1* and *AFB1* RNA levels, while expression of *AFB2* to *AFB5* remained unaffected (Fig. 5R).

We then monitored the *TIR1* and *AFB1* expression in wild-type, *jlo-2*, *jlo-5*, and *jlo-7* mutant embryos and roots using transcriptional and translational reporter lines (Fig. 5A–P; Supplementary Fig. S5A–N). *TIR1* and *AFB1* are broadly expressed throughout the root meristem of wild-type plants from embryogenesis onwards (*n*≥35; Fig. 5A, C, E, G, I, M and Supplementary Fig. S5A, D, G, J; Parry *et al.*, 2009). In comparison, we found strongly reduced *TIR1* and *AFB1* expression in homozygous *jlo-2*, *jlo-5*, and *jlo-7* mutant roots, both at the transcriptional and protein level (*n*≥35, Fig. 5B, D, F, H, K, O and Supplementary Fig. S5).

**Fig. 6.**
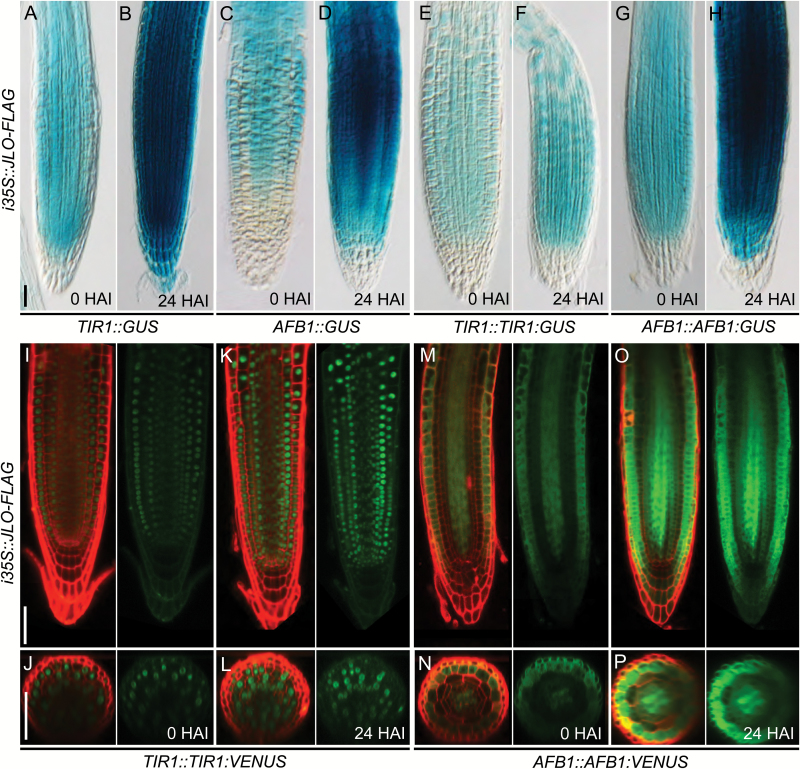
*JLO* misexpression rapidly induces *TIR1* and *AFB1* expression. Estradiol-inducible expression of a FLAG tagged JLO protein up-regulated expression levels of *TIR1* and *AFB1* in roots. (A, B) *TIR1::GUS* and (C, D) *AFB1::GUS* transcriptional reporter, (E, F) *TIR1::TIR1:GUS*, and (G, H) *AFB1::AFB1:GUS* translational reporter at 0 or 24 h after estradiol-induced (HAI) expression of *JLO-FLAG* (I–L) *TIR1::TIR1:VENUS* and (M–P) *AFB1::AFB1:VENUS* translational reporter at 0 or 24 HAI. (J, L, N, P) Root cross-sections at approximately 250 μm from the tip. Fluorescent reporter lines were stained with PI (red), VENUS expression in green. All seedlings were analysed at 5 days after germination. JLO–FLAG expression was induced with 20 μM β-estradiol. Scale bars: 50 μm.

Root cross-sections at approximately 250 μm from the tip of *TIR1::TIR1-VENUS* (Wang *et al.*, 2016) showed broad *TIR1* expression in all root cell layers (*n*=15, Fig. 5J), which was down-regulated in *jlo-2* (*n*=15, Fig. 5L). *AFB1* is predominantly expressed in epidermal cells and the procambium (Fig. 5N), and expression levels are strongly reduced in all cell types in the *jlo-2* mutant (Fig. 5P). To analyse if JLO is not only required for wild-type *TIR1* and *AFB1* expression levels, but also sufficient to up-regulate *TIR1* and/or *AFB1*, we used an estradiol-inducible *JLO* transgene (Bureau *et al.*, 2010). Induced expression of a JLO–FLAG fusion protein in wild-type roots was sufficient to cause a 3.6-fold up-regulation of *TIR1* RNA levels within 2 h after induction, and a 2.4-fold up-regulation of *AFB1* transcript levels. The *AFB2* to *AFB5* RNA levels were not affected by induced JLO misexpression (see Supplementary Fig. S6Q). Analysis of the reporter lines revealed that upon *JLO* induction, *TIR1* and *AFB1* were expressed in their normal patterns, but at higher levels (Fig. 6A–P and Supplementary Fig. S6). However, unlike wild-type plants, cells of the lateral root cap show *TIR1* expression (*n*≥35, Fig. 6B, F, K and Supplementary Fig. S6A–H). Furthermore, root cross-sections of *TIR1::TIR1-VENUS* disclosed increased *TIR1* expression in endodermal and vascular tissues (*n*=15, Fig. 6K, L). Likewise, the *AFB1* expression domain extended from the procambium to the adjacent vascular and ground tissues (Fig. 6D, H, O and Fig. 5I–P). Together these results suggest that of the six *TIR1*/*AFB* genes, JLO primarily promotes *AFB1* and *TIR1* expression during root development.

Given that *TIR1* expression is strongly reduced in *jlo* mutants, we asked if the reduction in *TIR1* levels can be causal for the *jlo* mutant phenotypes. We therefore generated double mutants between *jlo-2* and *tir1-1* (Ruegger *et al.*, 1998) and studied their genetic interaction (see Supplementary Table S4). Segregation analysis revealed no significant enhancement of the *jlo-2* mutation by *tir1-1* under normal growth conditions, and *jlo-2;tir1-1* double mutants were phenotypically indistinguishable from *jlo-2* single mutants (Fig. 5Q). Consistent with previously published results, the synthetic auxin analogue 2,4-D induces root stunting in wild-type, while *tir1-1* mutant roots are resistant and elongate their roots when grown on media containing 0.2 µM 2,4-D (Fig. 5B; Ruegger *et al.*, 1998). Similarly, *jlo-2/+* roots were less sensitive to the effects of 2,4-D (Fig. 5Q). Elimination of *TIR1* function from *jlo-2/+* mutants further decreased the auxin response, measured as root length reduction. However, the observed auxin response was similar in both *jlo-2* and *jlo-2;tir1-1* mutants (Fig. 5Q). This indicates that loss of *TIR1* function does not further affect auxin responses when the seedling is already lacking *JLO* function.

Taking the above data together, we hypothesized that JLO acts as an upstream transcriptional regulator of *TIR1* and *AFB1* expression in the auxin signaling cascade. The short root phenotype of *jlo* mutants is then partly due to an auxin insensitivity caused by the lack of TIR1 and AFB1. To test this, we expressed the coding sequences of *TIR1* and *AFB1* in Arabidopsis wild-type and *jlo-2* mutant seedlings from the cauliflower mosaic virus 35S promoter (*CaMV35S*). F3 progeny of Arabidopsis wild-type and *jlo-2* mutant seedlings that carry *2x35S::TIR1-FLAG* and *2x35S::AFB1-FLAG* transgenes were examined at 5 DAG.

**Fig. 7.**
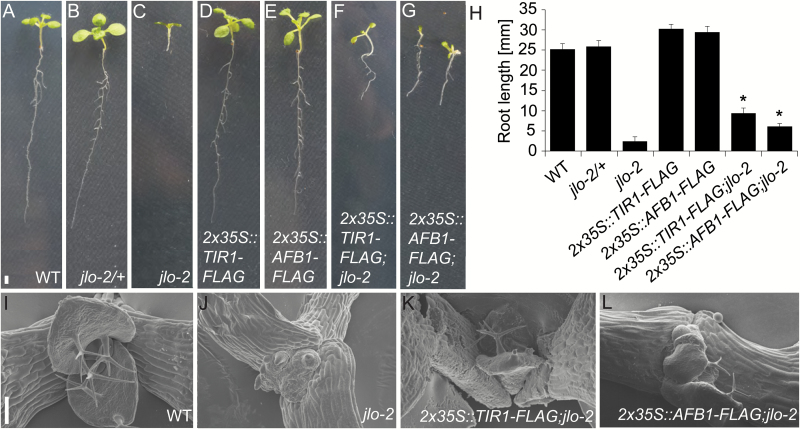
*JLO*-independent transgenic expression of *TIR1* or *AFB1* can rescue *jlo-2* mutant phenotypes. Seedlings 5 days after germination of (A) wild-type, (B) *jlo-2/+*, (C) *jlo-2*, (D) *2x35S::TIR1-FLAG*, (E) *2x35S::AFB1-FLAG*, (F) *2x35S::TIR1-FLAG; jlo-2*, and (G) *2x35S::AFB1-FLAG*; *jlo-2*. (H) Root length in millimetres of the indicated seedling genotypes. (I–L) Scanning electron micrographs of (I) wild-type, (J) *jlo-2*, (K) *2x35S::TIR1-FLAG*; *jlo-2*, and (L) *2x35S::AFB1-FLAG*; *jlo-2* shoots. All seedlings were 5 days old. Asterisks mark a significant difference from *jlo-2* mutant (*P≤0.01, analysed by Student’s *t*-test). Scale bars: 1 mm in (A–G) and 50 μm in (I–L). Error bars indicate standard error.

Constitutive expression of *TIR1* or *AFB1* did not change the phenotype during vegetative development of wild-type (Fig. 7D, E, H). At 5 DAG, wild-type roots are 30.7 ± 0.28 mm (*n*=48) long, while *jlo*-2 roots remain short (2.8 ± 0.16 mm, *n*=53, Fig. 7C, H; Bureau *et al.*, 2010). The *2x35S::TIR1-FLAG* and *2x35S::AFB1-FLAG* transgenes in *jlo-2* mutant background caused an increase in root growth to 6.9 ± 0.5 mm and 5.5 ± 0.31 mm, respectively (*n*≥57, Fig. 7F, G, H). Thus, *JLO*-independent expression of either *TIR1* or *AFB1* was sufficient to at least partially restore *jlo-2* root development.

Interestingly, transgenic expression of *TIR1* and *AFB1* also supported extended shoot development of *jlo-2*. The *jlo-2* homozygous mutants show a severe retardation in shoot growth, and mutant meristems initiate primordia at arbitrary positions (Fig. 7J; Rast and Simon, 2012) that either fail to grow out or develop into radialized organs (Fig. 7J). By 25 DAG, the *jlo-2* shoot meristems had stopped further growth. Constitutive expression of *TIR1* or *AFB1* in *jlo-2* mutants caused the formation of several small leaves carrying trichomes by 5 DAG (Fig. 7K, L) before leaf development and meristem activity eventually arrested within 25 DAG. We conclude that *JLO* is required for *TIR1* and *AFB1* expression during root and shoot development, and that the lack of auxin receptors is causal for at least some of the developmental defects observed in *jlo* mutants. To assess whether JLO directly regulates the expression of *TIR1* and *AFB1*, we tested whether JLO binds the *TIR1* and *AFB1* promoters using chromatin immunoprecipitation (ChIP)–quantitative PCR (qPCR) assays with seedling tissue. However, no significant enrichment for the *TIR1* or *AFB1* promoter, or for other intronic or exonic regions of these genes was detected. This indicates that the interaction of JLO to its target sequences is only very transient, or that JLO regulates *TIR1* and *AFB1* expression in an indirect manner (see Supplementary Fig. S6).

## Discussion

Auxin is a central regulator of plant growth and development, and the main function of auxin, the control of auxin responsive gene expression, relies on the TIR1/AFB (TRANSPORT INHIBITOR RESPONSE1/AUXIN SIGNALING F-BOX) clade of auxin receptors, which facilitate the degradation of the AUX/IAA transcriptional repressors in response to auxin (reviewed in Mockaitis and Estelle, 2008). Whilst the regulation of auxin synthesis, transport, and AUX/IAA degradation has been well studied, much less is known about the control of TIR1/AFB levels and activities, which can significantly contribute to overall auxin signaling (Dreher *et al.* 2006). In seedlings, TIR1 is an intrinsically unstable protein, and changes in TIR1 availability can influence auxin signaling. Furthermore, environmental factors such as ambient temperature strongly impact plant growth, which is partially mediated by auxin. Increased temperature was found to control the expression of auxin biosynthetic genes via PHYTOCHROME INTERACTING FACTOR4, but also to promote rapid accumulation of the TIR1 auxin co-receptor, an effect that is dependent on the molecular chaperone HEAT SHOCK PROTEIN 90 (Yu *et al.*, 2015; Wang *et al.*, 2016). Besides protein stability, auxin receptors can also be regulated post-transcriptionally through miR393 in response to pathogen attacks (Navarro *et al.*, 2006; Parry *et al.*, 2009).

Our study of JLO function and its mutant phenotypes now uncovers the importance of transcriptional regulation of *TIR1* and *AFB1*. *JLO* was first identified as an important developmental regulator in a large mutagenesis screen using transposable elements as activation tags. In that study, altered expression of *JLO* was shown to drastically affect organ initiation, leaf, and root development, and shoot and root meristem maintenance (Borghi *et al.*, 2007, Rast and Simon, 2012). Some of these effects could be assigned to the misexpression of *KNOX* genes in organ primordia and later during leaf development, since failure to maintain *KNOX* silencing affects acquisition of proper organ cell fate, and consequently also organ architecture and shape (Tsiantis *et al.*, 1999, Byrne *et al.*, 2000, Hay *et al.*, 2006). This indicates that *JLO* can regulate meristematic gene functions. *KNOX* down-regulation at the sites of lateral organ initiation coincides spatially and temporally with the establishment of a concentration maximum for auxin at the peripheral zone of the SAM, which depends on the auxin efflux carrier PIN1 (Benková *et al.*, 2003; Reinhardt *et al.*, 2003). Compromising PIN1 activity or auxin signaling results in ectopic expression of the *KNOX* gene *KNAT1* in Arabidopsis leaves (Hay *et al.*, 2006). Mutants in *KNAT1* can at least partially rescue the loss of lateral organs in *pin1* mutants, suggesting that a failure to repress *KNAT1* expression in the periphery of the SAM in *pin1* mutants could antagonize lateral organ formation (Hay *et al.*, 2006). These observations indicate antagonistic interactions between auxin signaling and *KNOX* gene expression or function, and *JLO* appears to play a role in regulating both of these processes. Importantly, early *jlo* mutant defects such as developmental arrest during embryonic or the first seedling stages could not be explained by misregulation of *KNOX* genes, but phenotypically resembled those found in mutants for key auxin signaling components (e.g. BDL/MP) (Bureau and Simon, 2008; Bureau *et al.*, 2010).

In previous studies (Borghi *et al.*, 2007; Bureau *et al.*, 2010), *JLO* was shown to act by promoting expression of several auxin responsive genes, for instance the auxin efflux facilitators *PIN.* However, the molecular mechanisms underlying *PIN* regulation was not known. We now show that *JLO* positively regulates the expression of auxin co-receptors *AFB1* and *TIR1*, and that the developmental defects observed in *jlo* mutants can be partially suppressed by constitutive transgenic, and therefore *JLO*-independent, expression of *AFB1* or *TIR1*. However, we were unable to detect direct binding of JLO to the promoter regions of *TIR1* or *AFB1*, suggesting that JLO controls *AFB1* and *TIR1* expression in an indirect manner. Furthermore, the expression domains of *JLO* and *TIR1* or *AFB1* overlap only partially in the RAM, indicating that JLO may act very early in development to establish *TIR1* and *AFB1* expression, which could be maintained at later stages independently of JLO. Importantly, *JLO*-dependent repression of the *KNOX* gene *KNAT1* in organ primordia had been previously shown to involve the MYB-class transcription factor AS1 and the LBD protein AS2 (Phelps-Durr *et al.*, 2005; Hay *et al.*, 2006; Guo *et al.*, 2008; Rast and Simon, 2012). AS1 and AS2 interact directly to repress *KNAT1* expression and bind the promoters of *KNAT1* and *KNAT2*, possibly as a repressive chromatin complex through recruitment of the histone chaperone histone regulatory protein A (HIRA) or members of the POLYCOMB REPRESSIVE COMPLEX 2 (PRC2) (Phelps-Durr *et al.*, 2005; Guo *et al.*, 2008; Lodha *et al.*, 2013). Thus, LBD family transcription factors could act transiently to direct chromatin modifiers to their targets. JLO was shown to physically interact with the AS2–AS1 heteromer via direct contacts with AS2 (Rast and Simon, 2012), and the strong mutant phenotype of *jlo* loss of function alleles could indicate that JLO acts transiently as a hub that assembles diverse repressive (and activating) chromatin modifying complexes. An initial survey of further protein–protein interactions within the LBD family via *in planta* FRET analysis showed that JLO and many other LBD proteins can form a range of different complexes (Berckmans *et al.*, 2011). Which types of complexes are formed in a given cell will then mainly depend on the specific subset of LBD proteins that are being expressed, and on their relative amount. The overall developmental context may then also determine the ultimate function of JLO, acting either as a transcriptional activator (of auxin receptors) or repressor (of *KNOX* genes).

Together, our data show that already from early embryogenesis onwards, *JLO* is required for expression of the auxin receptors TIR1 and AFB1, and thereby plays a central role in patterning processes throughout plant development. Thus, *JLO* can regulate auxin perception and consequently auxin transport and auxin-responsive gene expression. Differential regulation of specific auxin receptors may also provide an effective mechanism for adaptation of plants in response to changing environmental factors. We now need to uncover how *JLO* itself is controlled at the transcriptional level or via interaction with cofactors in order to gain fresh insights on the regulation of development in plants.

## Supplementary data

Supplementary data are available at *JXB* online.

Fig. S1. ChIP–qPCR shows no evidence for a direct interaction of JLO protein with the *TIR1* or *AFB1* loci.

Fig. S2. Phenotypic analysis of *jlo* mutants.

Fig. S3. Genetic interaction between *JLO* and the *PLT* genes.

Fig. S4. *AUX1* expression is reduced in *jlo-2*.

Fig. S5. *TIR1* and *AFB1* expression is down-regulated in *jlo* mutants.

Fig. S6. *TIR1* and *AFB1* expression changes after inducing *JLO-FLAG* expression.

Table S1. Oligonucleotides used for qRT-PCR analyses.

Table S2. Oligonucleotides used for ChIP-qPCR analyses.

Table S3. Genetic interactions between *JLO* and *PLT*-family genes.

Table S4. Genetic interactions between *JLO* and *TIR1*.

## Accession numbers

Sequence data from this article can be found in the Arabidopsis Genome Initiative or GenBank/EMBL databases under the following accession numbers: AT4g00220 (*JLO*), AT1g04550 (*BDL*), AT1g19850 (*MP*), AT3G62980 (*TIR1*) and AT4G03190 (*AFB1*).

## Author contributions

The authors have made the following declaration about their contributions. Conceived and designed the experiments: MRS, PZ, and RS. Performed the experiments: MRS, PZ, and SS. Analysed the data: MRS, PZ, and RS. Shared material and discussed the results: SK and MK. Wrote the paper: MRS, PZ, and RS.

## Supplementary Material

Supplementary_Figures_S1_S6

Supplementary_Tables_S1_S4
