# Supplementary material for: The Arabidopsis JAGGED LATERAL ORGANS (JLO) gene sensitizes plants to auxin
Source: J Exp Bot. 2017 May 2;68(11):2741–55. doi: 10.1093/jxb/erx131 (PMC5853575; doi:10.1093/jxb/erx131)
Supplement: Supplementary_Figures_S1_S6 [file erx131_suppl_supplementary_figures_s1_s6.pdf]

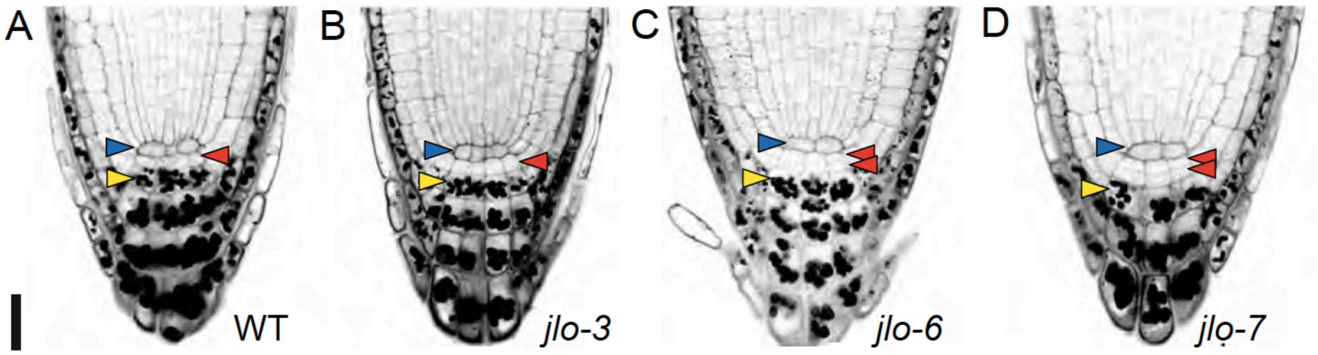

Supplemental Figure 1: Phenotypic analysis of *jlo*-mutants

(a-d) Differentiation status of distal root meristem in (a) wildtype, (b) *jlo-3,* (c) *jlo-6* and (d) *jlo-7* roots, mPSPI stained. CSCs (red arrowheads), quiescent center (blue arrowheads), columella cells (yellow arrowheads).

All seedlings were 5 days old and grown on Murashige Skoog (MS) plant media.

Scale bar: 50 µm.

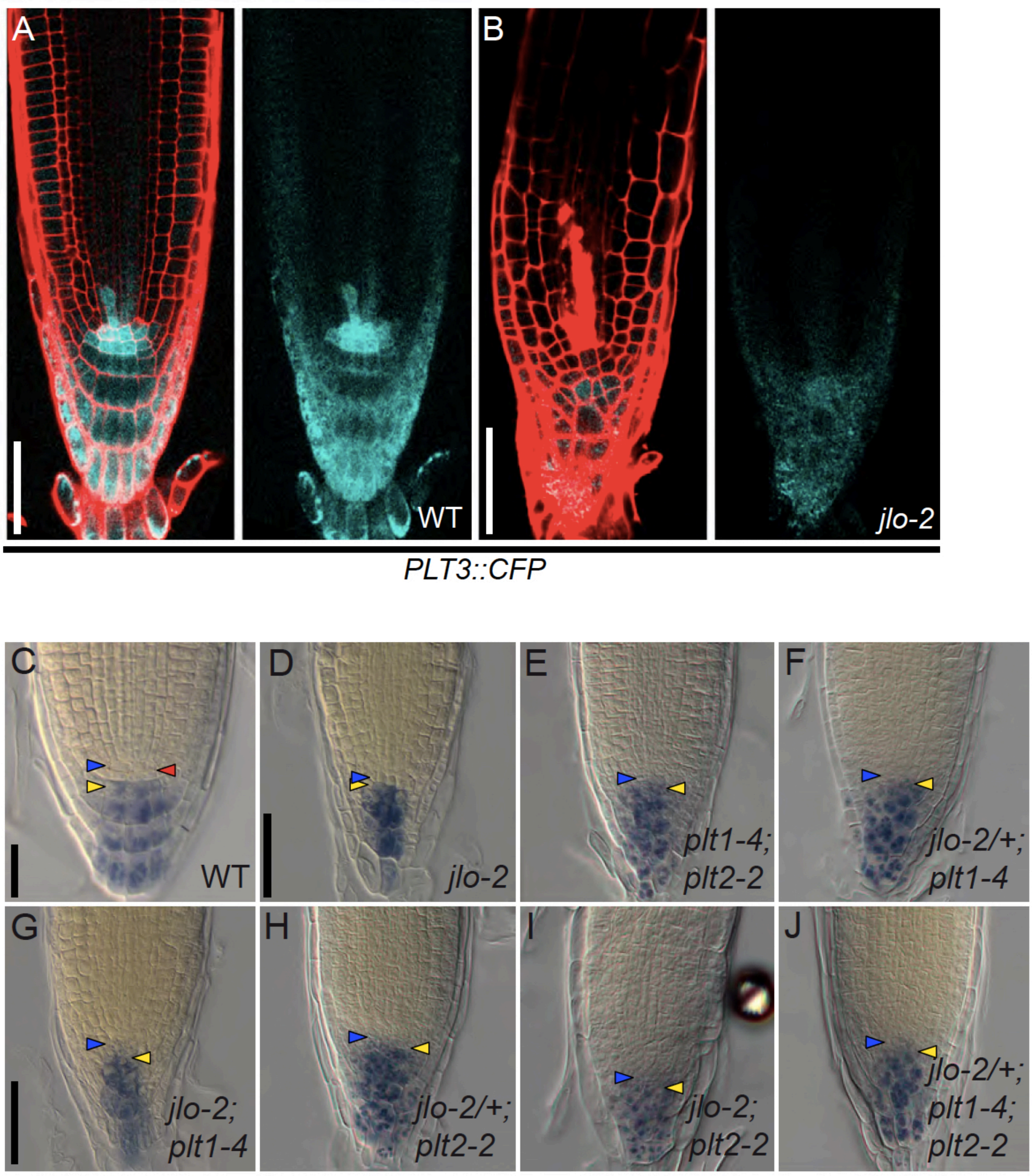

Supplemental Figure 2: Genetic interaction between *JLO* and the *PLT* genes

(a,b) Expression of a *PLT3::CFP* transcriptional reporter line in (a) wildtype and (b) *jlo-2* roots, stained with PI. (d-j) Differentiation status in the distal root meristems of (c) wildtype, (d) *jlo-2*, (e) *plt1-4 plt2-2,* (f) *jlo-2/+ plt1-4,* (g) *jlo-2 plt1-4,* (h) *jlo-2/+ plt2-2,* (i) *jlo-2 plt2-2* and (j) *jlo-2/+ plt1-4 plt2-2*. Lugol staining was used to visualize starch granules in differentiated columella cells. Red arrow: QC, Blue arrow: CSCs, lacking stainable starch granules; Yellow arrow: first differentiated columella cells. All seedlings were 5 days old. WT: wildtype; Scale bars: 50 µm.

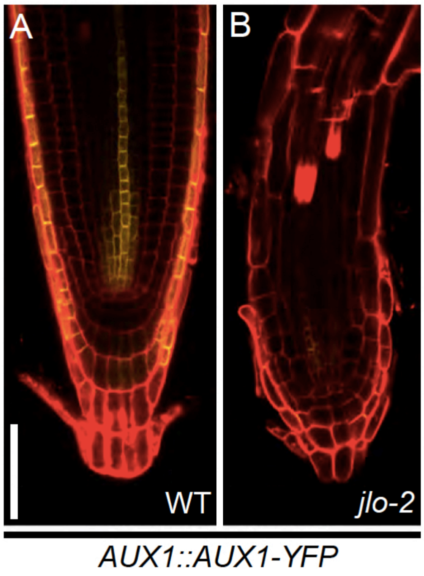

Supplemental Figure 3: *AUX1* expression is reduced in *jlo-2*

(a,b) Expression of a *AUX1::AUX1:YFP* translational reporter line in (a) wildtype and (b) *jlo-2* , stained with PI. All seedlings were 5 days old. WT: wildtype; Scale bars: 50 µm.

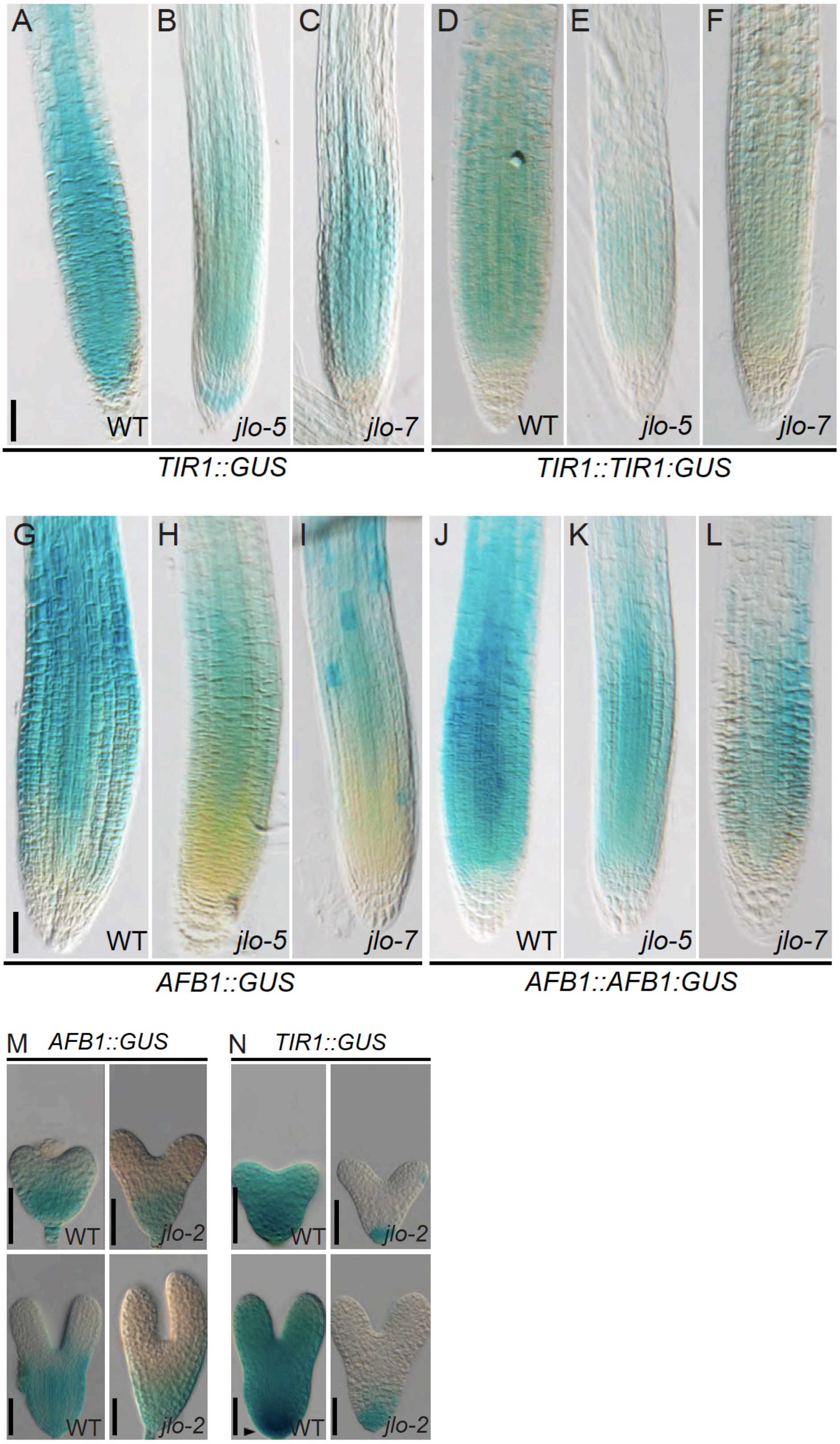

Supplemental Figure 4: *TIR1* and *AFB1* expression is downregulated in *jlo* mutants

(a-c) Expression of *TIR1::GUS,* (d-f) *TIR1::TIR1:GUS,* (g-i) *AFB1::GUS* and (j-l) *AFB1::AFB1:GUS* in root meristems of wt, *jlo-5* or *jlo-7*, respectively. All seedlings were 5 days old. (m,n) *AFB1::GUS* or *TIR1::GUS* expression in wt or *jlo-2* mutant

embryos at heart stage (tope row) or torpedo stage (bottom row). Black triangle in (n) highlight expression peak in the developing root meristem.

WT: wildtype. Scale bars: 50 µm.

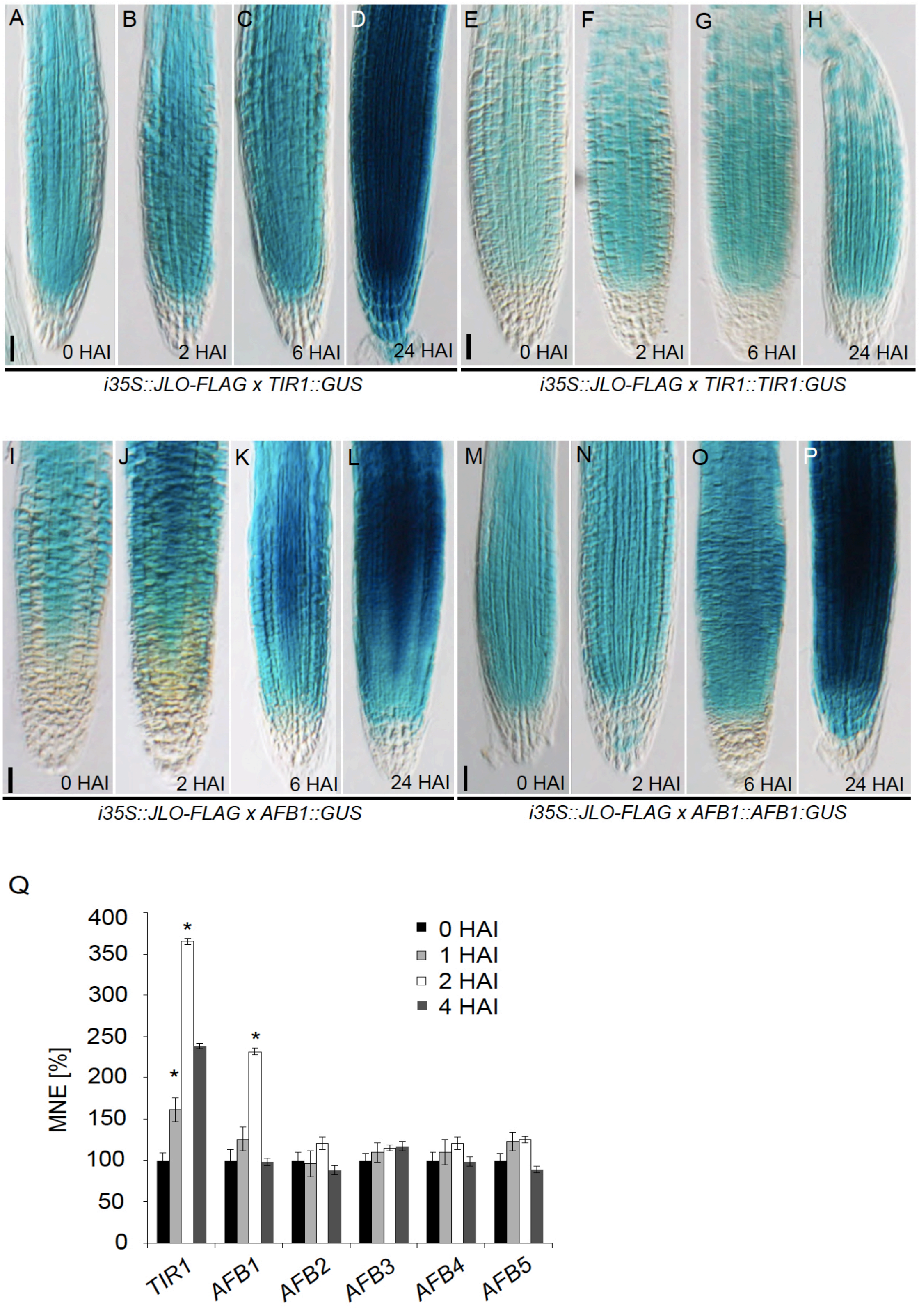

Supplemental Figure 5: *TIR1* and *AFB1* expression changes after inducing *JLO-FLAG* expression

Roots expressing (a-d) *TIR1::GUS,* (e-h) *TIR1::TIR1:GUS,* (i-l) *AFB1::GUS* or (m-p), *AFB1::AFB1:GUS* at 0 to 24 hours after induction (HAI) of JLO-FLAG expression. (q) qRT-PCR analysis of *TIR1* and *AFB1* to *5* RNA levels upon induction of JLO-FLAG

expression by 20μM β-estradiol. Expression levels were normalized to uninduced controls prepared at the same time points. All seedlings were 5 days old. MNE: mean normalized expression; Asterisks mark a significant difference from uninduced transgene JLO-FLAG ($p \leq 0.01$*; analyzed by Student's t-test). Scale bar: 50μm. Error bars indicate standard error.

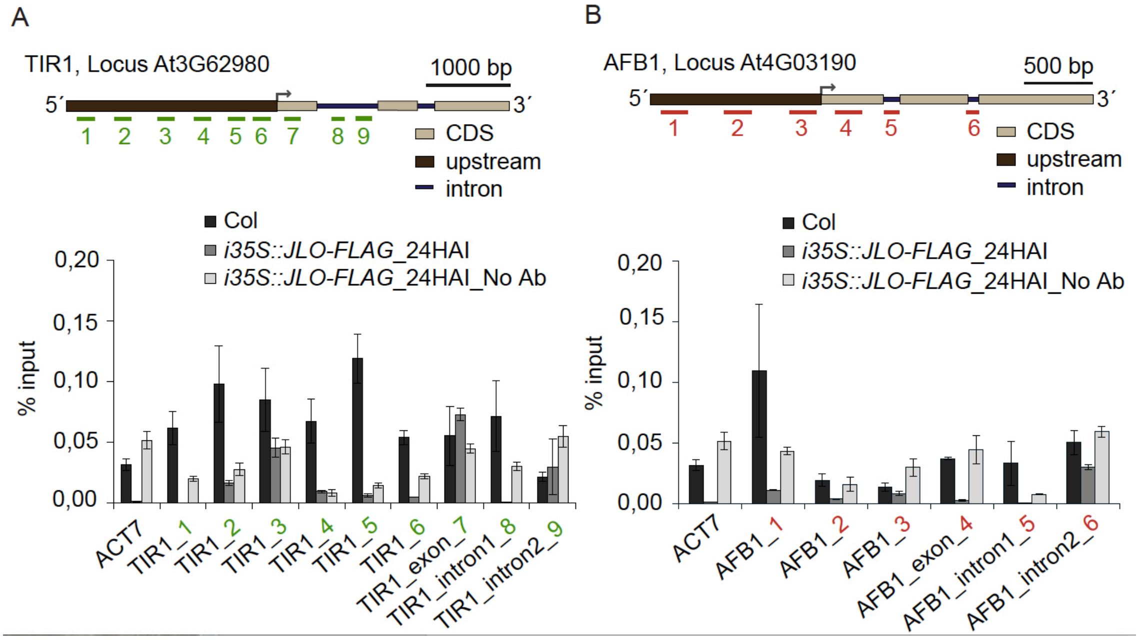

Supplemental Figure 6: ChIP-qPCR shows no evidence for a direct interaction of JLO protein with the *TIR1* or *AFB1* loci

(a,b) Chromatin-immunoprecipitated DNA analysis by quantitative real-time PCR**.** Schematic representations of the *TIR1* and *AFB1* loci are shown at the top. The PCR-amplified regions of *TIR1 (*green dashes) (a) and *AFB1* (red dashes) (b) are diagramed in the upper panel; transcription start site (arrow); ORFs (light brown boxes); regions upstream (5´) of ATG (dark brown boxes) and introns (thin lines). Anti-FLAG antibody was used for the analysis, and “no antibody (no Ab)” served as the negative control. Whole seedlings were used for ChIP assay. Results normalised relative to input.

No Ab: no antibody. Error bars indicate standard error.
