# Supplementary material for: The Arabidopsis JAGGED LATERAL ORGANS (JLO) gene sensitizes plants to auxin
Source: J Exp Bot. 2017 May 2;68(11):2741–55. doi: 10.1093/jxb/erx131 (PMC5853575; doi:10.1093/jxb/erx131)
Supplement: Supplementary_Tables_S1_S4 [file erx131_suppl_supplementary_tables_s1_s4.pdf]

Parental genotype	N	% WT	% mutant	phenotype (%)				*p*-value
				jlo-2 like	no root	no root/ hypocotyl	Σ	
WT	400	100	0	-	-	-	0	
plt1-4	341	100	0	-	-	-	0	
plt2-2	378	100	0	-	-	-	0	
plt1-4 plt2-2	312	100	0	-	-	-	0	
jlo-2/+	405	84,0	16,0	16,0	-	-	16,0	
jlo-2/+ plt1-4	441	85,0	15,0	14,8	0,2	-	15,0	0,8
jlo-2/+ plt2-2	547	84,5	15,5	15,3	0,2	-	15,5	0,9
jlo-2/+ plt1-4 plt2-2	543	85,5	14,5	8,5	5,1	0,9	14,5	0,8

Supplemental table1:
Genetic interactions between JLO and PLT-family genes

**Parental genotype**	Progeny phenotype in % (N)			
	N	WT	*jlo-2* like	*p*-value
WT	321	100% (321)	-	
tir1-1	156	100% (156)	-	
jlo-2/+	240	86% (206)	14% (34)	
jlo-2/+;tir1-1	207	87% (180)	13% (27)	0,8

Supplemental Table 2:
Genetic interactions between JLO and TIR1

Oligonucleotides used for qPCR analyses

anotation	locus	sequence forward primer	sequence reverse primer
TIP41-like	At4g34270	GTGAAAACTGTTGGAGAGAAGCAA	TCAACTGGATACCCTTTCGCA
WOX5	At3g11260	AATATATTCGAGAGACCACCACGAC	CTAATGGCGGTGGATGTTCC
SCR	At3g54220	TTCTTCTACCGATGCACCACC	AAAGCCTCCGCCGTATTTG
SHR	At4g37650	GCAAACGGAGCAATCTTGGA	TTGAGTGCAAAACGTGGAGC
PLT1	At3g20840	CTATTATCCCATAGATGAGCCT	ACAACGAACCTCGATCTAT
PLT2	At1g51190	AAAAGTAGCGGATTCTCGCG	CCTTGCTTGCCATCTTCCAT
PLT3	At5g10510	CTGTTTTGATCTGGCCGCTC	TCGAGGGTGACACCGAAAG
BBM	At5g17430	GTAGATACGAGGCACATTTATGG	TGTCATAACCTCCTTGTCTTCC
MP	At1g19850	GCCATATCTACCGAGGGCAACCAA	GACTTCTCATCCCTGATGAACAAAACA
BDL	At1g04550	GGCTTTTAGATGGATCATCAGACTTTGT	AGTTGATAAACATTCTCCATGGAACATC
TIR1	At3g62980	TCTTGTGCTTTCTTCCTGCGAAGG	AGCTCTTTCAGATTCCTGCAAGTGG
AFB1	At4g03190	CAGCTACTTGCAGGAACTTGAGAGTGT	CTGGAAAATAGCTAAGCCAATCTCCTC
AFB2	At3g26810	GCTGAGATTCATGGTAGCCACCTTATT	CCTATACTATCCAAAATCCATAACCGCT
AFB3	At1g12820	CTAATTGCAGGCATCTTCGTGAGCTG	TGGAAAACAGTTCAGCCATTGACCTC
AFB4	At4g24390	TGCTCAAGCCCATCATAAGCAAC	TCGAGTCAAGAGCCCAGAAGACTC
AFB5	At5g49980	CAGGGAAACAAGAGATCCAATCCACTT	TTGCCAACAAGTGCAGAAAGCTGA

Supplemental Table 3:
Oligonucleotides used for qRT-PCR analyses

Oligonucleotides used for ChIP-qPCR analyses

ACTIN7, locus At5g0981		
primer name	sequence forward primer	sequence reverse primer
ACT7	CCAGGAATTGCTGACCGTAT	GGTGCAACCACCTTGATCTT

TIR1, locus: At3g62980, promoter region (2446 bp from TTS)		
primer name	sequence forward primer	sequence reverse primer
TIR1_1	TCCACTAATGGGGAAGGTCA	GCATGTGTGGACGGACATA
TIR1_2	AGCCATGCGTGGGATTATTA	GGGAGAAGTGTGGAGCTGAG
TIR1_3	AAGAAGGGCATTCTCGTCAA	CAGATCTGGGACCCCTTTTT
TIR1_4	TCCAATCTCACACGAAAACACT	CCCACCACTTGGATTCATAA
TIR1_5	CGTGCATCGCGTATCATTAG	CAAAATAGCGCGAGAAGGAG
TIR1_6	TCCTTCCCCCTATCCTTTTC	AGATCTGAGGCGCCATTAAC

TIR1, locus: At3g62980, exon region		
primer name	sequence forward primer	sequence reverse primer
TIR1_exo	GGTGTGCAAGTCATGGTACG	ACGAAGACATGGCCTCAATC

TIR1, locus: At3g62980, intron region		
primer name	sequence forward primer	sequence reverse primer
TIR1_int1	TTTTCGGATTCCCCTTCTTT	TTAGCATCGAGTGACCCACA
TIR1_int2	CATACATTAACACATGAATTTGGAA	GAGCACAGTTACAAAAAGAAAAAG

AFB1, locus: At4g03190, promoter region (1238 bp from TTS)		
primer name	sequence forward primer	sequence reverse primer
AFB1_1	TAGAGGATTCGCCATTCGAG	TCATAGACGCTATTCCGCTAAA
AFB1_2	TGTTAACTGACGCGGTAAACTG	TCAACGTTTTTGGACGTGAA
AFB1_3	ATGGGTGCAAGCTCAACTTT	GGGGGAAGAAATGAAGAAAAA

AFB1, locus: At4g03190, exon region		
primer name	sequence forward primer	sequence reverse primer
AFB1_exo	CCGGAACTCTGTTTCTCTGG	ATAACCACCCCAACCATCAG

AFB1, locus: At4g03190, intron region		
primer name	sequence forward primer	sequence reverse primer
AFB1_int1	TCGGTTCCAACCAAATACAA	TTGAAAATCAGACAACAACAAAAA
AFB1_int2	AACTGCAGAAGCTATGGGTGA	TTGCAATATGAGGCAACAGC

Supplemental Table 4:
Oligonucleotides used for ChIP-qPCR analyses
